# Rapid decline of yearly number of hip arthroscopies in Sweden: a retrospective time series of 6,105 hip arthroscopies based on a national patient data register

**DOI:** 10.1080/17453674.2021.1928396

**Published:** 2021-05-21

**Authors:** Tobias Wörner, Frida Eek, Jesper Kraus-Schmitz, Mikael Sansone, Anders Stålman

**Affiliations:** 1 Department of Health Sciences, Lund University, Lund; 2 Capio Artro Clinic, Stockholm; 3 Stockholm Sports Trauma Research Center, Department of Molecular Medicine and Surgery, Karolinska Institutet, Stockholm; 4 Department of Orthopaedics, Skåne University Hospital, Malmö; 5 Gothenburg Sports and Trauma Research Center, Institute of Clinical Sciences, Sahlgrenska Academy, University of Gothenburg, Gothenburg, Sweden

## Abstract

Background and purpose — Hip arthroscopies (HAs) have increased exponentially worldwide and are expected to continue rising. We describe time trends in HA procedures in Sweden (10 million inhabitants) between 2006 and 2018 with a focus on procedure rates, surgical procedures, and patient demographics such as age and sex distribution.

Patients and methods — We retrospectively collected data from the Swedish National Patient Register (NPR) for all surgeries including surgical treatment codes considered relevant for HA from 2006 to 2018. Surgical codes were validated through a multiple-step procedure and classified into femoroacetabular impingement syndrome (FAIS) related or non-FAIS related procedure. Frequencies, sex differences, and time trends of surgical procedures and patient demographics are presented.

Results — After validation of HA codes, 6,105 individual procedures, performed in 4,924 patients (mean age 34 years [SD 12]) were confirmed HAs and included in the analysis. Yearly HA procedure rates increased from 15 in 2006 to 884 in 2014, after which a steady decline was observed with 469 procedures in 2018. The majority (65%) of HAs was performed in males. Male patients were younger, and surgeries on males more frequently included an FAIS-related procedure.

Interpretation — Similar to previous studies in other parts of the world, we found dramatic increases in HA procedures in Sweden between 2006 and 2014. Contrary to existing predictions, HA rates declined steadily after 2014, which may be explained by more restrictive patient selection based on refined surgical indications, increasing evidence, and clinical experience with the procedure.

Hip arthroscopy was long deemed impossible due to anatomic constraints. Easier arthroscopic access to knee and shoulder joints led to an increasing arthroscopy rate in these joints during the 1990s and 2000s (Kim et al. 2011, Colvin et al. 2012a). During the 1990s, improved surgical equipment and techniques enabled surgeons to gain easier access to the hip joint for diagnosis and treatment of a variety of pathologies (Griffiths and Khanduja 2012), including femoroacetabular impingement syndrome (FAIS), acetabular labrum tears, and chondral lesions (Bedi et al. 2013). Arthroscopic hip surgery has been one of the fastest emerging fields within orthopedics and might be at a tipping point for even wider use (Khan et al. 2016a).

An exponential worldwide increase in performed HAs has been documented between 2000 and 2013, based on data from private insurance databases (Sing et al. 2015, Maradit Kremers et al. 2017, Bonazza et al. 2018), performance data from surgical trainees (Colvin et al. 2012b, Bozic et al. 2013) and data from national health services (Palmer et al. 2016). While exponentially more patients received HA, evidence for its effectiveness has been questioned (Reiman and Thorborg 2015). In recent years, RCTs have indicated that hip arthroscopy may be more effective than structured rehabilitation in the treatment of FAIS (Griffin et al. 2018, Palmer et al. 2019). The clinical relevance of the statistical superiority for HA found in these trials is debated (Ferreira et al. 2021); however, a continued rise in HA rates has been predicted worldwide (Khan et al. 2016a, Palmer et al. 2016). The only study assessing HA rates beyond 2013 reports declining rates in Finland after 2014 (Karelson et al. 2020). In Sweden, time trends regarding HA have not been investigated. It is therefore unknown whether the rise in HA has continued, or if surgical practice has changed over the years.

Therefore, we describe frequency and time trends in performance of hip arthroscopies, with regards to performance rates, surgical procedures, and patient demographics (age and gender distribution) in Sweden.

## Patients and methods

### Study design

A retrospective analysis of national patient register data was undertaken.

### Data source

Data was retrieved from the Swedish National Patient Register (NPR). The NPR is a national register, established by the National Board of Health and Welfare in 1960. Since 2001 all in- and outpatient services in Sweden are obliged to provide patient, geographical, administrative, as well as medical data to the NPR. Surgical procedures in the NPR are coded according to the Swedish version of the NOMESCO Classification of Surgical Procedures (NCSP-S). Each surgery can contain several different surgical codes. Diagnoses are coded according to the International Classification of Diseases (ICD), version 10.

### Data collection and data validation

During the data collection we applied a multiple step procedure to validate the data and to yield a final cohort of surgeries with best possible specificity.

### Step 1: Initial data request from the NPR

We requested data for all surgeries including any NCSP-S codes potentially indicating HA, that started with NE (“Musculoskeletal system, Pelvis”) or NF (Musculoskeletal system, Hip joint and thigh”) and were performed between 2001 and 2018. The collected data for each surgery included all surgical codes as well as diagnostic codes, clinic, date, patient age, and sex.

### Step 2: Primary selection and classification based on national coding practice

Next, we categorized surgical codes and selected cases according to coding practices, which, as agreed upon during meetings of Swedish orthopedic surgeons performing HA, were expected to be used nationally. We thus selected all cases with surgical codes that potentially represented HA. Surgeries with combinations of codes including simultaneous codes for hip replacement surgery were excluded.

### Step 3: Validation of codes through personal communication with clinics

Contrary to our expectations, we did not find uniform coding practices but large variation between clinics. We therefore presented all surgical units known to perform HA (N = 18) with the preliminary results for their clinic and asked them to verify number of performed HAs and codes used. Data for all clinics that, to our knowledge, were not performing HA were excluded. In case of discrepancies between coding practices applied in our data set and coding practice reported by the clinics, we re-categorized the codes in our data set to fit actual applied practice. During this process we discovered that the 2 clinics with highest general HA rates had a complete gap in reporting (1 and 2.5 years respectively). Therefore, we requested and received the unavailable data directly from these clinics. With updated coding practices and previously unavailable data added to the file we then categorized codes as: FAIS surgery (cam resection, pincer resection, unspecified FAIS surgery), or non-FAIS surgery (psoas tenotomy, cartilage procedure, synovectomy, removal of loose bodies, labral procedures).

### Step 4: Final selection after exclusion and qualitative review of selected cases

Surgeries with diagnosis codes indicating simultaneous fracture, or surgical codes indicating knee surgery, were excluded from the data set. In the last step, an orthopedic surgeon (AS) reviewed all surgeries with any HA code and excluded surgeries with unrealistic code combinations or other indications of open surgery as opposed to arthroscopic procedures. Due to few potential HAs and uncertain coding during the 1st years of the period, we finally included, and report on, surgeries performed between 2006 and 2018.

### Ethics, funding, and potential conflicts of interest

This study was approved by the ethical board of Karolinska Institute (Dnr: 2019-04514). We received no external funding for the performance of the study. While 3 of the authors are clinically involved with performing HA or treating patients following the procedure, none declare any conflicts of interest that could affect the results of this study.

## Results

**Figure 1.**
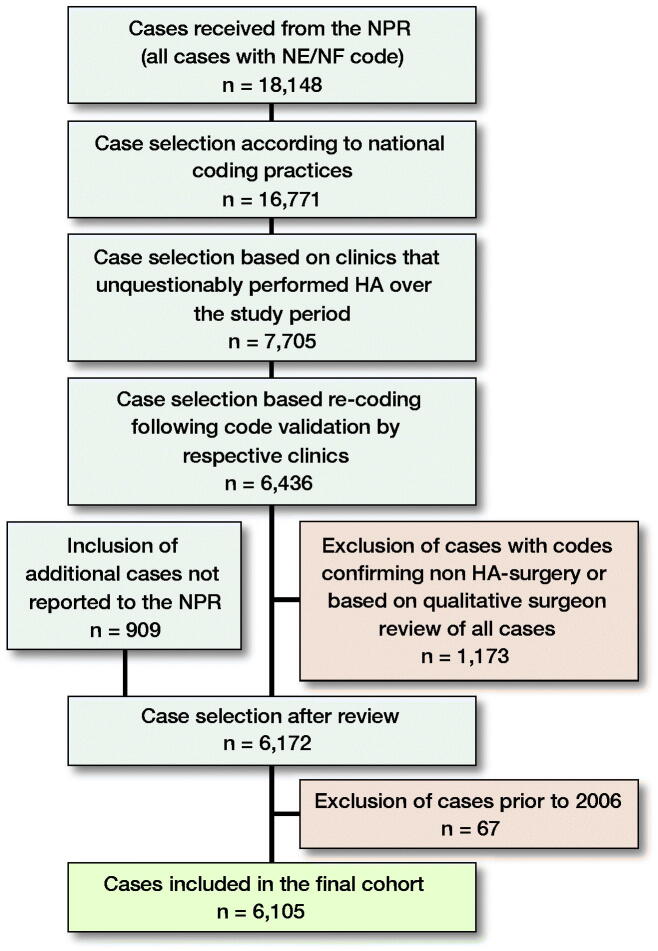
Flowchart of the selection and validation process.

We received 18,148 individual cases with any NE or NF surgical codes from the NPR. Each surgery case included between 1 and 30 unique surgical codes. After the selection and validation process, 6,105 individual procedures were included in the final analysis (Figure 1). The 6,105 final procedures were performed in 4,924 individual patients (Table). The majority of HAs (65%) were performed on male patients. Mean age (SD) at first surgery was 34 years (SD 12). The majority of patients (82%) had only 1 HA, 15% had two HAs, and 4% had 3 or more HAs performed during the study period. NPR data excludes information concerning the side of surgery, therefore we could not differentiate between reoperations and bilateral surgery in cases where surgery was performed on different occasions.

**Table.** Procedures performed among all identified hip arthroscopies (n = 6,105). Values are count (%)

FAIS surgery	5,226 (86)	
	with unspecific surgical codes	4,127 (68)
	with specific surgical codes	1,099 (18)
	Cam resection	946 (16)
	Pincer resection	279 (5)
Non-FAIS-related surgery ^a^	879 (14)	
	Psoas tenotomy	367 (6)
	Synovectomy	243 (4)
	Removal of free body	78 (1)
	Diagnostic arthroscopy	127 (2)
	Labral reconstruction	64 (1)
Cartilage procedures	4,924 (81)	
	During FAIS surgery	4,402 (72)
	During non-FAIS-related surgery	522 (9)
Number of HA codes in same surgery	1	1,335 (22)
	2	4,500 (74)
	3	252 (4)
	4	18 (0.3)

FAIS = femoroacetabular impingement syndrome.

a Non-FAIS-related procedures may occasionally have been performed during FAIS related surgery.

**Figure 2.**
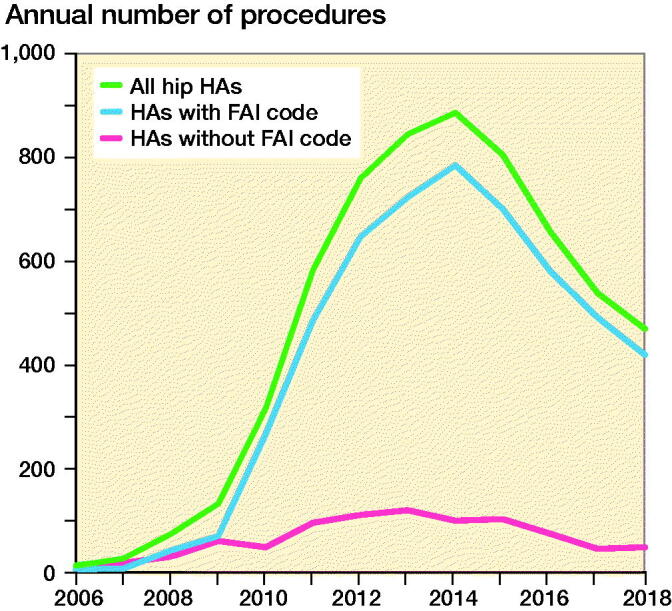
Number of hip arthroscopies (HAs) performed between 2006 and 2018. FAI = Femoroacetabular impingement.

**Figure 3.**
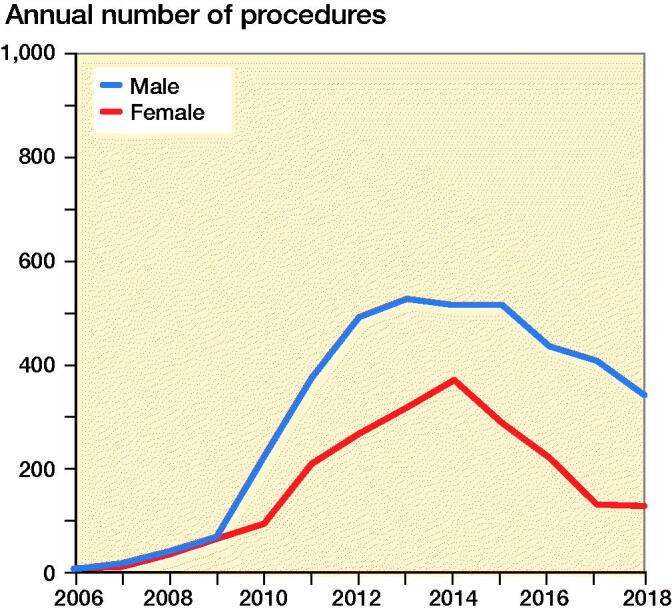
Number of hip arthroscopies between 2006 and 2018 by sex.

**Figure 4.**
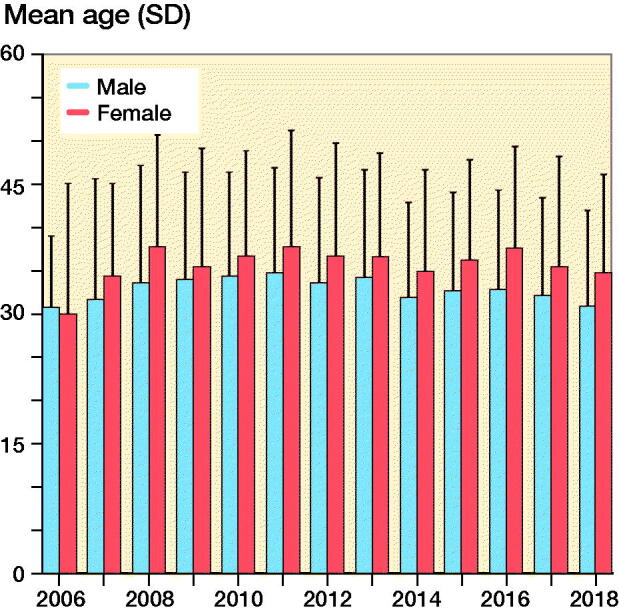
Age at time of hip arthroscopy for men and women.

A FAIS procedure was included in 90% of HAs performed on male and 77% of HAs performed on female patients. The total number of performed HAs increased from 15 in 2006 to 884 in 2014, after which it declined steadily to 469 in 2018 (Figure 2). During the first years, equal proportions of males and females received HA; however, from 2009 onwards, men comprised 60–70% of the patients undergoing the procedure (Figure 3). Average age at the time of surgery remained between 30 years (SD 12) and 36 years (SD 13) years throughout the study period. Male patients were generally younger (mean 33 years [SD 12]) than female patients (mean 36 years [SD 12]) (Figure 4).

## Discussion

This retrospective analysis of a national patient registry describes an exponential increase in arthroscopic procedures with treatment codes for hip arthroscopy between 2006 and 2014. The increase in HA rates appears to be driven by an increase in diagnosis and treatment of FAIS, which was more often performed in male than in female patients. After 2014, procedure rates of HA began to drop every year, with this decrease continuing until the end of the study period in 2018.

### Hip arthroscopy in Sweden between 2006 and 2014

Hip arthroscopy gained popularity in the early 2000s and its uptake increased rapidly during the first decade of the new millennium (Colvin et al. 2012b, Bozic et al. 2013, Sing et al. 2015, Palmer et al. 2016, Maradit Kremers et al. 2017, Bonazza et al. 2018). We observed the same pattern in Sweden with few procedures in 2006, an exponential increase in HA rates between 2008 and 2012, and the peak in 2014. The life cycle of a surgical technique can be described in several stages (McCulloch et al. 2009), which have also been discussed in the context of HA practice (Khan et al. 2016a). After an innovation stage, in which a new treatment is used by pioneering surgeons as a solution to a clinical problem, the treatment is developed and explored further by early adopters, and larger numbers of patients thus receive surgery with broadened indications. FAIS-related procedures (cam and pincer resection) were the driving indications behind the increased HA rates in our study. In 2003, surgeons from Switzerland coined the terms cam and pincer morphology, reporting observed impingement between articular surfaces leading to femoroacetabular impingement (Ganz et al. 2003). This mechanical phenomenon is suggested to be a cause for symptoms and an etiological factor for the development of osteoarthritis (Ganz et al. 2003), which serves as theoretical framework for surgical treatment for FAIS (Griffin et al. 2018). FAIS has been defined by a recent consensus as a clinical disorder with a triad of typical symptoms, clinical findings, and radiological evidence of cam and/or pincer morphology (Griffin et al. 2016). Between 2005 and 2010 the number of scientific publications (mainly case series and expert opinion) on arthroscopic treatment of FAIS increased rapidly. During this period, which can be considered the development and exploration stage of HA in the treatment of FAIS, we observed the most rapid increase in HA rates in Sweden. Our data shows a continued increase in rates between 2010 and 2014, a time during which the number of scientific publications on FAIS continued to increase, including an increasing number of prospective studies (Khan et al. 2016b). During the same period, national and local hip arthroscopy registries were developed in Denmark and Sweden (Sansone et al. 2014, Mygind-Klavsen et al. 2016).

### Hip arthroscopy in Sweden between 2014 and 2018

Our study is one of the first studies to describe HA rates beyond 2013. In the 1st years following the peak in 2014, HA rates declined steeply, but this decline was less marked towards 2018. A similar pattern has been observed in a recent study from Finland, where HA rates declined after a peak in 2013 (Karelson et al. 2020). Due to the lack of comparable studies from other parts of the world we do not know if HA rates follow a similar pattern in other countries during this time period or if they keep on rising as predicted by previous studies (Khan et al. 2016a, Palmer et al. 2016). The decline in surgery rates may be explained by the natural development of surgical practice after its innovation, development, and exploration stage (McCulloch et al. 2009). In the exploration phase, a new technique is adopted by increasing numbers of surgeons and indications for the procedure are explored and broadened. Through a prospective learning process based on surgical outcomes, surgeons likely refine their surgical indications over time. This increased awareness of patient selection, potentially facilitated by 1st results of register-based studies (Sansone et al. 2014, Mygind-Klavsen et al. 2016), may be a potential explanation for the decrease in HA rates after 2014.

HA practice can be considered to have only been on the verge of the assessment stage of surgical innovation (McCulloch et al. 2009) once the number of performed procedures started to decline. HA rates in Sweden and Finland started to decline 4 years before the first randomized trials tested effectiveness of the procedure in comparison with non-surgical treatments (Griffin et al. 2018, Palmer et al. 2019). Based on our data we can only judge the development of HA procedure rates until 2018 and are therefore not able to identify potential effects of emerging RCT evidence. This evidence points towards superior outcomes in patients with FAIS when treated using HA compared with non-surgical treatment (Ferreira et al. 2021). It is reasonable to assume that more RCT evidence, better knowledge about which patients benefit most from HA (e.g., increased treatment effect for resection of cam morphology) (Griffin et al. 2018), and improved non-surgical treatment strategies will lead to more evidence-based clinical practice. In turn, HA rates may reach a steady level in coming years.

### Surgical procedures and patient demographics

The rates of performed HA observed in our study were driven by FAIS-related surgery. We also found that 2/3 of all HA were performed on male patients, which is in contrast to previous database studies where the majority of patients were female (Palmer et al. 2016, Maradit Kremers et al. 2017, Bonazza et al. 2018). We believe that these 2 findings are related to each other and reflect the Swedish approach to HA. The main indication for HA is resection of cam and pincer morphology with the aim to treat FAIS. Resection of cam morphology is the most frequently performed HA procedure in Sweden (Sansone et al. 2014). This is also in line with our data, which shows that cam resections were 3 times more prevalent than pincer resections among all procedures with a specific FAIS code. Cam morphology is far better understood than pincer morphology. Cam morphology develops during adolescence and there is a dose–response relationship with athletic activity (Palmer et al. 2018). Further, cam morphology is associated with future risk of hip osteoarthritis (Agricola et al. 2013) and the treatment effect of removing a cam morphology is likely greater than that of removing a pincer morphology (Griffin et al. 2018). Cam morphology is found to be more common in men than in women (van Klij et al. 2018). This, in combination with the Swedish approach, focusing on the resection of cam morphology as primary indication for HA, may offer a possible explanation for our finding that the majority of patients receiving HA were men. With a mean age of 35 at the time of index HA, patients in our study were also younger than patients in previous studies (> 40 years) (Maradit Kremers et al. 2017, Bonazza et al. 2018). In our study, male patients receiving HA were also younger (mean 33 years) than female patients (mean 37 years) receiving HA. The age and sex distribution observed in our study is, however, similar to other Swedish studies on HA patients (Sansone et al. 2014). As judged by our data, relatively young male patients treated for FAIS and likely receiving cam resection are the primary group of patients undergoing HA in Sweden.

### Methodological considerations

While previous studies exploring time trends in HA have predominantly been based on data sources such as insurance data sets (Sing et al. 2015, Maradit Kremers et al. 2017, Bonazza et al. 2018), databases for surgical trainee performance (Colvin et al. 2012b, Bozic et al. 2013), or national health services excluding the private sector (Palmer et al. 2016), our study is based on a population-wide patient registry including all surgeries performed in Sweden during the study period. Due to inconsistency and variability in national coding practice in our data set we took extra measures in validating treatment codes and improving interpretability of our data. Through direct contact with surgical units and feedback regarding individual coding practices, we believe we have reached high specificity of our final selection of cases. Only a small share of FAIS-related procedures used specific surgical codes indicating cam or pincer resection, leaving us no other choice than coding many procedures under the umbrella term FAIS surgery. Furthermore, we did not perform a review of patient journals to confirm the individual procedures that have been coded.

## Conclusion

The number of HA procedures performed in Sweden increased exponentially between 2006 and 2014. After 2014, HA rates declined steadily until 2018. The rise and fall of HA rates appear to be driven by treatment for FAIS, which is most frequently performed on male patients.

## References

[CIT0001] Agricola R, Heijboer M P, Bierma-Zeinstra S M, Verhaar J A, Weinans H, Waarsing J H. Cam impingement causes osteoarthritis of the hip: a nationwide prospective cohort study (CHECK). Ann Rheum Dis 2013; 72(6): 918–23. 22730371 10.1136/annrheumdis-2012-201643

[CIT0002] Bedi A, Kelly B T, Khanduja V. Arthroscopic hip preservation surgery: current concepts and perspective. Bone Joint J 2013; 95-B(1): 10–9. 23307667 10.1302/0301-620X.95B1.29608

[CIT0003] Bonazza N A, Homcha B, Liu G, Leslie D L, Dhawan A. Surgical trends in arthroscopic hip surgery using a large national database. Arthroscopy 2018; 34(6): 1825–30. 29580743 10.1016/j.arthro.2018.01.022

[CIT0004] Bozic K J, Chan V, Valone F H, 3rd, Feeley B T, Vail T P. Trends in hip arthroscopy utilization in the United States. J Arthroplasty 2013; 28(8 Suppl.): 140–3. 23916639 10.1016/j.arth.2013.02.039

[CIT0005] Colvin A C, Egorova N, Harrison A K, Moskowitz A, Flatow E L. National trends in rotator cuff repair. J Bone Joint Surg Am 2012a; 94(3): 227–33. 22298054 10.2106/JBJS.J.00739 PMC3262185

[CIT0006] Colvin A C, Harrast J, Harner C. Trends in hip arthroscopy. J Bone Joint Surg Am 2012b; 94(4): e23. 22336982 10.2106/JBJS.J.01886

[CIT0007] Ferreira G E, O’Keeffe M, Maher C G, Harris I A, Kwok W S, Peek A L, Zadro J R. The effectiveness of hip arthroscopic surgery for the treatment of femoroacetabular impingement syndrome: s systematic review and meta-analysis. J Sci Med Sport 2021; 24(1): 21–9. 32616421 10.1016/j.jsams.2020.06.013

[CIT0008] Ganz R, Parvizi J, Beck M, Leunig M, Notzli H, Siebenrock K A. Femoroacetabular impingement: a cause for osteoarthritis of the hip. Clin Orthop Relat Res 2003; (417): 112–20. 10.1097/01.blo.0000096804.78689.c2 14646708

[CIT0009] Griffin D R, Dickenson E J, O’Donnell J, Agricola R, Awan T, Beck M, Clohisy J C, Dijkstra H P, Falvey E, Gimpel M, Hinman R S, Holmich P, Kassarjian A, Martin H D, Martin R, Mather R C, Philippon M J, Reiman MP, Takla A, Thorborg K, Walker S, Weir A, Bennell K L. The Warwick Agreement on femoroacetabular impingement syndrome (FAI syndrome): an international consensus statement. Br J Sports Med 2016; 50(19): 1169–76. 27629403 10.1136/bjsports-2016-096743

[CIT0010] Griffin D R, Dickenson E J, Wall P D H, Achana F, Donovan J L, Griffin J, Hobson R, Hutchinson C E, Jepson M, Parsons N R, Petrou S, Realpe A, Smith J, Foster N E, FASHIoN Study Group. Hip arthroscopy versus best conservative care for the treatment of femoroacetabular impingement syndrome (UK FASHIoN): a multicentre randomised controlled trial. Lancet 2018; 391(10136): 2225–35. 29893223 10.1016/S0140-6736(18)31202-9 PMC5988794

[CIT0011] Griffiths E J, Khanduja V. Hip arthroscopy: evolution, current practice and future developments. Int Orthop 2012; 36(6): 1115–21. 22371112 10.1007/s00264-011-1459-4 PMC3353094

[CIT0012] Karelson M C, Jokihaara J, Launonen A P, Huttunen T, Mattila V M. Lower nationwide rates of arthroscopic procedures in 2016 compared with 1997 (634925 total arthroscopic procedures): has the tide turned? Br J Sports Med 2020. Online ahead of print. 10.1136/bjsports-2019-101844 PMC8408579 32241819

[CIT0013] Khan M, Ayeni O R, Madden K, Bedi A, Ranawat A, Kelly B T, Sancheti P, Ejnisman L, Tsiridis E, Bhandari M. Femoroacetabular impingement: have we hit a global tipping point in diagnosis and treatment? Results from the InterNational Femoroacetabular Impingement Optimal Care Update Survey (IN FOCUS). Arthroscopy 2016a; 32(5): 779–87 e4. 26775733 10.1016/j.arthro.2015.10.011

[CIT0014] Khan M, Oduwole K O, Razdan P, Phillips M, Ekhtiari S, Horner N S, Samuelsson K, Ayeni O R. Sources and quality of literature addressing femoroacetabular impingement: a scoping review 2011–2015. Curr Rev Musculoskelet Med 2016b; 9(4): 396–401. 27628053 10.1007/s12178-016-9364-5 PMC5127944

[CIT0015] Kim S, Bosque J, Meehan J P, Jamali A, Marder R. Increase in outpatient knee arthroscopy in the United States: a comparison of National Surveys of Ambulatory Surgery, 1996 and 2006. J Bone Joint Surg Am 2011; 93(11): 994–1000. 21531866 10.2106/JBJS.I.01618

[CIT0016] Maradit Kremers H, Schilz S R, Van Houten H K, Herrin J, Koenig K M, Bozic K J, Berry D J. Trends in utilization and outcomes of hip arthroscopy in the United States between 2005 and 2013. J Arthroplasty 2017; 32(3): 750–5. 27793498 10.1016/j.arth.2016.09.004

[CIT0017] McCulloch P, Altman D G, Campbell W B, Flum D R, Glasziou P, Marshall J C, Nicholl J, Balliol C, Aronson J K, Barkun J S, Blazeby J M, Boutron I C, Campbell W B, Clavien P A, Cook J A, Ergina P L, Feldman L S, Flum D R, Maddern G J, Nicholl J, Reeves B C, Seiler C M, Strasberg S M, Meakins J L, Ashby D, Black N, Bunker J, Burton M, Campbell M, Chalkidou K, Chalmers I, de Leval M, Deeks J, Ergina P L, Grant A, Gray M, Greenhalgh R, Jenicek M, Kehoe S, Lilford R, Littlejohns P, Loke Y, Madhock R, McPherson K, Meakins J, Rothwell P, Summerskill B, Taggart D, Tekkis P, Thompson M, Treasure T, Trohler U, Vandenbroucke J. No surgical innovation without evaluation: the IDEAL recommendations. Lancet 2009; 374(9695): 1105–12. 19782876 10.1016/S0140-6736(09)61116-8

[CIT0018] Mygind-Klavsen B, Gronbech Nielsen T, Maagaard N, Kraemer O, Holmich P, Winge S, Lund B, Lind M. Danish Hip Arthroscopy Registry: an epidemiologic and perioperative description of the first 2000 procedures. J Hip Preserv Surg 2016; 3(2): 138–45. 27583150 10.1093/jhps/hnw004 PMC5005047

[CIT0019] Palmer A J, Malak T T, Broomfield J, Holton J, Majkowski L, Thomas G E, Taylor A, Andrade A J, Collins G, Watson K, Carr A J, Glyn-Jones S. Past and projected temporal trends in arthroscopic hip surgery in England between 2002 and 2013. BMJ Open Sport Exerc Med 2016; 2(1): e000082. 10.1136/bmjsem-2015-000082 PMC5117047 27900161

[CIT0020] Palmer A, Fernquest S, Gimpel M, Birchall R, Judge A, Broomfield J, Newton J, Wotherspoon M, Carr A, Glyn-Jones S. Physical activity during adolescence and the development of cam morphology: a cross-sectional cohort study of 210 individuals. Br J Sports Med 2018; 52(9): 601–10. 28798039 10.1136/bjsports-2017-097626 PMC5909766

[CIT0021] Palmer A J R, Ayyar Gupta V, Fernquest S, Rombach I, Dutton S J, Mansour R, Wood S, Khanduja V, Pollard T C B, McCaskie A W, Barker K L, Andrade T, Carr A J, Beard D J, Glyn-Jones S, FAIT Study Group. Arthroscopic hip surgery compared with physiotherapy and activity modification for the treatment of symptomatic femoroacetabular impingement: multicentre randomised controlled trial. BMJ 2019; 364:l185. 10.1136/bmj.l185 PMC6365841 30733197

[CIT0022] Reiman M P, Thorborg K. Femoroacetabular impingement surgery: are we moving too fast and too far beyond the evidence? Br J Sports Med 2015; 49(12): 782–4. 25677797 10.1136/bjsports-2014-093821

[CIT0023] Sansone M, Ahlden M, Jonasson P, Thomee C, Sward L, Baranto A, Karlsson J, Thomee R. A Swedish hip arthroscopy registry: demographics and development. Knee Surg Sports Traumatol Arthrosc 2014; 22(4): 774–80. Epub 2014 Jan 25. 24464406 10.1007/s00167-014-2840-9

[CIT0024] Sing D C, Feeley B T, Tay B, Vail T P, Zhang A L. Age-related trends in hip arthroscopy: a large cross-sectional analysis. Arthroscopy 2015; 31(12): 2307–13. 26194938 10.1016/j.arthro.2015.06.008

[CIT0025] van Klij P, Heerey J, Waarsing J H, Agricola R. The prevalence of cam and pincer morphology and its association with development of hip osteoarthritis. J Orthop Sports Phys Ther 2018; 48(4): 230–8. 29548271 10.2519/jospt.2018.7816

